# Stabilization of Self-Disintegrating Refined Ferrochrome Slags Using Desulfurized Borate Ore from the Inder Deposit: A Comparative Study with Colemanite

**DOI:** 10.3390/ma19142947

**Published:** 2026-07-09

**Authors:** Murat Kuanyshev, Otegen Sariyev, Nurbek Aitkenov, Zhumabay Tukashev, Bauyrzhan Kelamanov, Almas Yerzhanov, Gulnara Zhabalova, Aigerim Abilberikova

**Affiliations:** 1 Department of Transport Technology, Transportation Organization and Construction, K. Zhubanov Aktobe Regional University, Aktobe 030000, Kazakhstan; mkuanyshev@zhubanov.edu.kz; 2 Department of Metallurgy and Mining, K. Zhubanov Aktobe Regional University, Aktobe 030000, Kazakhstan; osariyev@zhubanov.edu.kz (O.S.); bkelamanov@zhubanov.edu.kz (B.K.); a.abilberikova@tttu.edu.kz (A.A.); 3 Department of Metallurgy and Materials Science, Karaganda Industrial University, Temirtau 101400, Kazakhstan; 4 Department of Energy, Karaganda Industrial University, Temirtau 101400, Kazakhstan

**Keywords:** refined ferrochrome slag, slag stabilization, borate ore, Ca_2_SiO_4_ transformation, boron oxide, autoclave testing, waste valorization, road construction materials, thermodynamic modeling, ferroalloy production

## Abstract

**Highlights:**

- RDBO effectively stabilizes self-disintegrating refined ferrochrome slags.
- Stable borate phases suppress β→γ-Ca_2_SiO_4_ transformation and cracking.
- RDBO-stabilized slags showed 25× lower mass loss than colemanite slags.
- Boron in RDBO remains bound in calcium borates, preventing metal contamination.
- Stabilized slag can be reused as a sustainable material for road construction.

**Abstract:**

The production of refined ferrochrome is accompanied by the formation of self-disintegrating slags caused by the β→γ-Ca_2_SiO_4_ polymorphic transformation during cooling, resulting in environmental and technological problems. This study aimed to develop and validate a technology for long-term stabilization of refined ferrochrome slags using remelted and desulfurized borate ore (RDBO) from the Inder deposit as an alternative to colemanite. Thermodynamic modeling, X-ray diffraction (XRD), scanning electron microscopy (SEM), and pilot-industrial trials were conducted to evaluate phase transformations, microstructure formation, and slag stability. The results showed that the addition of boron oxide promoted the formation of stable borate phases, primarily CaB_4_O_7_, and reduced the content of unstable Ca_2_SiO_4_ phases. Autoclave testing under saturated steam conditions (400 kPa, ~180 °C, 24 h) demonstrated complete preservation of structural integrity in all RDBO-stabilized samples, with an average mass loss of 0.19 ± 0.01%, whereas colemanite-stabilized slags exhibited cracking and partial disintegration. The stabilization effect was achieved at a B_2_O_3_ content of 0.17–0.47 wt.% without boron contamination of ferrochrome. The proposed technology ensures long-term slag stability and enables the utilization of stabilized slag as a technogenic material for road construction.

## 1. Introduction

The production of refined ferrochrome is accompanied by the formation of slags prone to self-disintegration during cooling, which leads to the generation of fine particulate matter and the formation of a significant environmental burden. Slag disintegration results in atmospheric air pollution and promotes the leaching of toxic components, including chromium compounds, into groundwater [1,2].

The primary cause of self-disintegration is the presence of unstable phases in the slag that undergo volumetric changes during cooling. These phases include, primarily, dicalcium silicate (Ca_2_SiO_4_), as well as forsterite, merwinite, and periclase [3,4,5,6]. The most critical transformation is the polymorphic transition β-Ca_2_SiO_4_→γ-Ca_2_SiO_4_, which is accompanied by a volume increase of 10–12%, leading to the development of internal stresses and the destruction of the slag structure [7].

One of the most widely used approaches to slag stabilization is the application of boron-containing additives. It has been established that the introduction of boron oxide (B_2_O_3_) and its compounds suppresses the polymorphic transformation of Ca_2_SiO_4_ through the formation of stable borate phases [8,9,10]. In industrial practice, natural borate minerals are widely used, particularly colemanite, which simultaneously reduces melt viscosity and contributes to the stabilization of slag structure [11,12,13,14].

At the Aktobe Ferroalloy Plant, raw colemanite from the Kestelek deposit (Türkiye) has been used for slag stabilization since 2020. However, this approach has several significant drawbacks. Colemanite contains impurities (Na, Cu, Sr, Mo, Pb, etc.) that negatively affect furnace lining durability and environmental safety [11,12,13,14]. In addition, when introduced into the furnace, part of the boron transfers to the metal, leading to contamination of ferrochrome and limiting its applications. Another important limitation is the temporary nature of stabilization: due to insufficient dosage and non-uniform distribution of the additive in the melt, the stabilized slag undergoes secondary disintegration after several months, forming dust-like products [15,16].

Previous attempts have been made to use borate ores from the Inder deposit (Western Kazakhstan) for slag stabilization [17]. However, the high sulfur content in the raw material (up to 14.9%) led to intensive SO_2_ emissions upon contact with the melt, rendering this approach unacceptable from the standpoint of industrial safety and environmental protection [18].

Despite the substantial body of research on the use of boron-containing additives for slag stabilization, several key challenges remain unresolved, including the temporary nature of stabilization, incomplete assimilation of additives, and boron contamination of the metal. Existing technological solutions do not provide a simultaneous resolution of these limitations under industrial conditions.

In this context, the development of an efficient stabilization technology capable of ensuring long-term structural stability of slag while preventing boron transfer to the metal represents a critical and relevant task.

The aim of this study is to develop and validate, under pilot-industrial conditions, a technology for the stabilization of self-disintegrating slags of refined ferrochrome using remelted and desulfurized borate ore (RDBO) from the Inder deposit. Particular attention is given to the analysis of phase transformations, microstructure formation, as well as the evaluation of long-term slag stability and its potential for subsequent utilization.

## 2. Materials and Methods

### 2.1. Characteristics of the Materials Used

Three types of boron-containing materials were used in this study (Table 1). Colemanite from the Kestelek deposit (Mustafakemalpaşa, Bursa, Türkiye) has been used at the Aktobe Ferroalloy Plant since 2020 as a stabilizer for refined ferrochrome (RFC) slags. The B_2_O_3_ content is approximately 38%. The charge per heat is 115 kg (≈1% of the tapped slag mass). The additive is introduced either into the furnace or into the tapping ladle.

Remelted borate ore (RDBO) from the Inder deposit (Western Kazakhstan) is a product obtained by melting borate ore followed by desulfurization. Its chemical composition (according to plant laboratory data) is presented in Table 1. The B_2_O_3_ content ranges from 11.12 to 14.01%, while the sulfur content is 0.46%. The particle size of the initial material is 50–150 mm. Fused borate ore (FBO) from the Inder deposit (Western Kazakhstan) was also evaluated. Its chemical composition is presented in Table 1. The sulfur content of the material was 14.9%, which was the main reason for its limited applicability in industrial trials. The required addition per heat was calculated to ensure the same amount of B_2_O_3_ as in the case of colemanite:

$$m_{RDBO}=\frac{115\;kg\times 38\%\;}{12.5\%}=350\;kg$$

### 2.2. Pilot-Industrial Test Conditions

**Figure 1:**
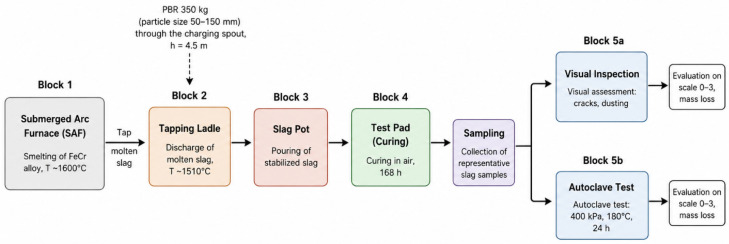
Process flow diagram of the pilot-industrial trials for the stabilization of refined ferrochrome (RFC) slag using processed borate ore (PBO).

Pilot-industrial trials were conducted at the Aktobe Ferroalloy Plant using a submerged arc furnace with a slag output of approximately 10 t per heat. The overall technological sequence of the trials is presented in Figure 1. Twenty-four hours prior to the start of the experiments, the addition of boron-containing additives was discontinued to eliminate residual B_2_O_3_ in the tapped slag.

Stage 1 (RDBO): Remelted borate ore was introduced into the tapping ladle during slag tapping from the furnace. The material was manually charged through a technological hatch at an elevation of 4.5 m. The addition per heat was 350 kg. A total of three heats were performed, and the resulting slag was poured into a slag pot.

Stage 2 (FBO): Fused borate ore was introduced in a similar manner with a charge of 590 kg (calculated based on a B_2_O_3_ content of 7.45%). Due to intense gas evolution and a pungent odor of sulfur oxides, the trials were discontinued.

### 2.3. Sampling and Analytical Methods

Slag samples were collected from the furnace prior to tapping and from the tapping ladle during slag discharge. The chemical composition was determined in the plant’s certified chemical laboratory (certificate No. KZ.0.05.0702) using X-ray fluorescence (XRF) and titrimetric analysis methods. X-ray diffraction (XRD) analysis of the slags was carried out using a Rigaku Ultima IV X-ray diffractometer (Rigaku Corporation, Tokyo, Japan) with Cu-Kα radiation (λ = 1.541874 Å) over a 2θ range of 5–90°, with a step size of 0.02° and a scanning rate of 5°/min. Phase identification of the diffraction patterns was performed using Portable Crystal Impact Match! software (Version 4.5, Crystal Impact, Bonn, Germany). Samples for XRD analysis were collected from slag pots after complete cooling of the slags to room temperature.

### 2.4. Evaluation of Slag Stability Against Disintegration

The long-term stability of slag was evaluated using two methods:

Visual observation—Slag was kept in a slag pot under ambient outdoor conditions for 168 h, followed by inspection for crack formation and dust generation.

Autoclave testing—This was conducted according to the procedure described in [19]. Slag samples with a particle size of 50–150 mm were cleaned with a brush to remove loose particles, washed, and dried to constant mass. The samples were then placed in molds and treated in an autoclave under saturated steam conditions. The pressure was increased to 400 kPa over 30 min, maintained for 24 h, and then reduced to atmospheric pressure over 20 min. After removal, the samples were visually examined for signs of disintegration (cracking, fragmentation).

Additionally, slag samples were collected after 1.5 months of storage at a slag disposal site and subjected to repeated autoclave testing.

### 2.5. Utilization Methods of Stabilized Slag

Until 2005, stabilized refined ferrochrome (RFC) slag treated with raw borate ore from the Inder deposit was routinely utilized in road construction. Based on accumulated industrial experience, road base compositions incorporating stabilized slag as a mineral binder were developed and patented. Following the transition to colemanite from the Kestelek deposit due to the unavailability of local borate raw materials, the practice of slag utilization in road construction was discontinued. The stabilization technology based on processed borate ore (PBO) developed in the present study provides the basis for restoring the large-scale utilization of RFC slag at the Aktobe Ferroalloy Plant (AFP).

## 3. Results

### 3.1. Thermodynamic Modeling

Thermodynamic modeling of phase equilibria was performed using the HSC Chemistry 10 software package. Equilibrium compositions were calculated using the Gibbs free energy minimization algorithm implemented in the “Equilibrium Composition” module over the temperature range of 25–2500 °C in a gaseous nitrogen atmosphere (2.24 m^3^) at a gas-phase pressure of 1 atm. The chemical composition of refined ferrochrome (RFC) slag (Table 2) was used as the initial system composition, with B_2_O_3_ additions ranging from 0 to 5 kg per tonne of slag.

The equilibrium temperatures were selected to cover the key stages of slag processing and phase transformations: 25 °C (storage conditions), 520 °C (the temperature range of the β→γ polymorphic transformation of dicalcium silicate responsible for slag disintegration), 1015 °C (melting temperature of borate phases), 1510 °C (RFC slag tapping temperature), and 2005 °C and 2500 °C to illustrate thermodynamic trends at elevated temperatures. It should be noted that HSC Chemistry calculates equilibrium among condensed and gaseous phases without employing a liquid slag model. Therefore, the results obtained at 2005 °C and 2500 °C should be interpreted as thermodynamic trends rather than the actual state of the molten slag.

**Figure 2:**
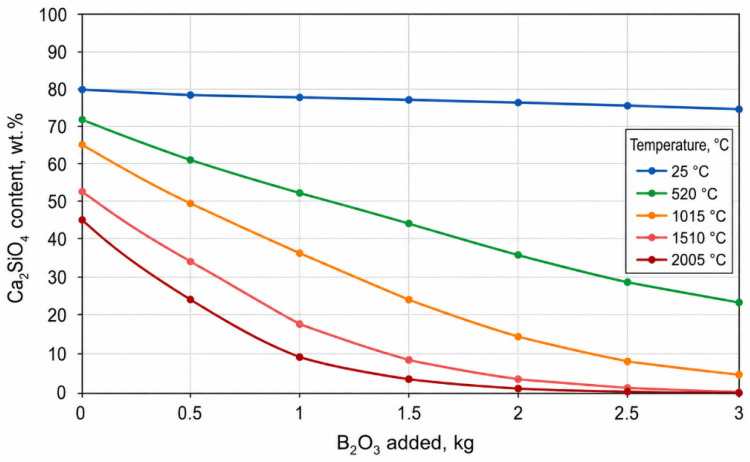
Dependence of Ca_2_SiO_4_ phase degradation on the amount of added B_2_O_3_.

**Figure 3:**
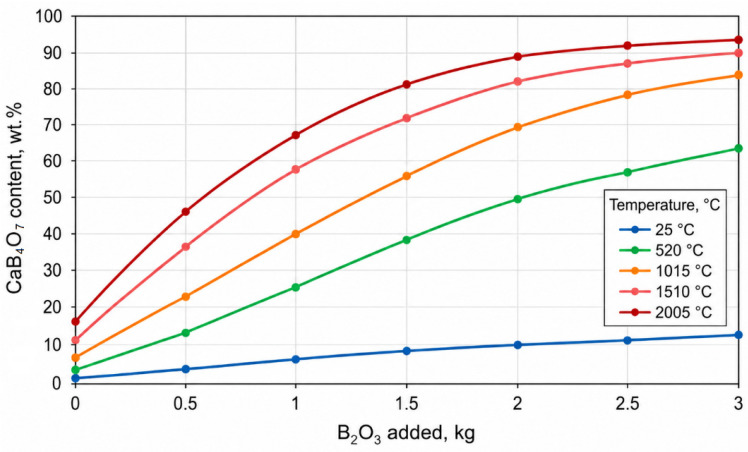
Effect of B_2_O_3_ addition on the formation of the stable borate phase (CaB_4_O_7_).

The results of phase equilibrium modeling in the “RFC slag—B_2_O_3_” system are presented in Table 3 and in Figure 2 and Figure 3. It was established that with increasing B_2_O_3_ content, borate phases are formed, predominantly calcium tetraborate (CaB_4_O_7_), accompanied by a systematic decrease in the content of β- and γ-Ca_2_SiO_4_, which are the main phases responsible for silicate disintegration.

At a B_2_O_3_ addition of 3.5 kg per ton of slag, the Ca_2_SiO_4_ content at temperatures below 1000 °C decreases by 20–25% compared to the initial composition, while the CaB_4_O_7_ content reaches 12–13%. These results confirm the fundamental possibility of slag stabilization through the binding of calcium oxide into stable borate complexes.

### 3.2. X-Ray Diffraction Analysis of Slags

**Figure 4:**
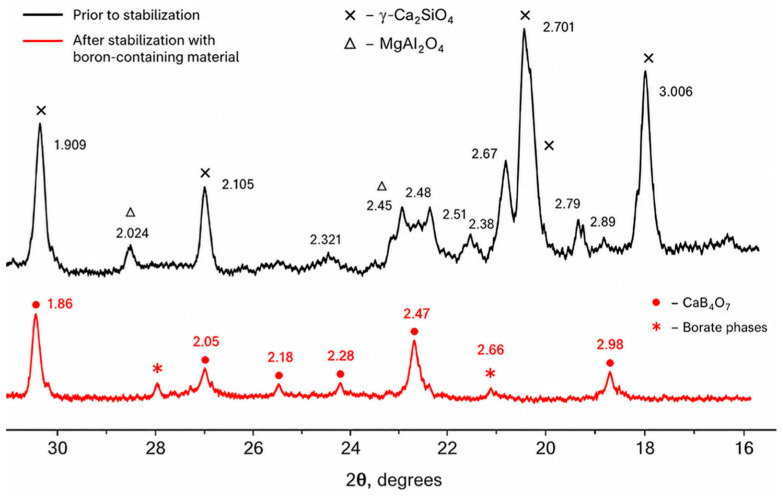
Comparative X-ray diffraction patterns of refined ferrochrome slags before and after stabilization.

To confirm the phase composition of slags obtained using different stabilization methods, X-ray diffraction (XRD) analysis was performed. The results are presented in Figure 4.

It was established that in the slag stabilized with remelted borate ore (RDBO), stable borate phases are formed, primarily calcium tetraborate (CaB_4_O_7_), along with a decrease in the intensity of peaks corresponding to dicalcium silicate (Ca_2_SiO_4_).

The obtained results are in good agreement with the thermodynamic modeling data (Section 3.1), confirming that the addition of boron oxide promotes the binding of CaO into stable borate compounds and reduces the amount of unstable silicate phases.

### 3.3. Chemical Composition of Slags with RDBO Addition

Table 4 presents the results of chemical analysis of slag samples taken from the furnace and from the tapping ladle during the addition of remelted borate ore (RDBO).

As can be seen from Table 4, in samples taken from the furnace (after discontinuation of colemanite addition), the B_2_O_3_ content is close to zero. In contrast, samples taken from the ladle, where RDBO was introduced, show B_2_O_3_ contents ranging from 0.01 to 0.21%. The relatively low values in some samples are attributed to insufficient contact time and the coarse particle size of RDBO (50–150 mm), which limited complete dissolution of the material in the molten slag.

After holding in the slag pot and at the disposal site, stabilized slag samples were collected, and their chemical composition is presented in Table 5.

The B_2_O_3_ content in the samples collected from the disposal site ranged from 0.17 to 0.47%, which in some cases is below the level of 0.35% recommended by the Ural Institute of Metals. However, as demonstrated by subsequent tests, this level was sufficient to prevent slag disintegration.

### 3.4. Visual Assessment of Stability

**Figure 5:**
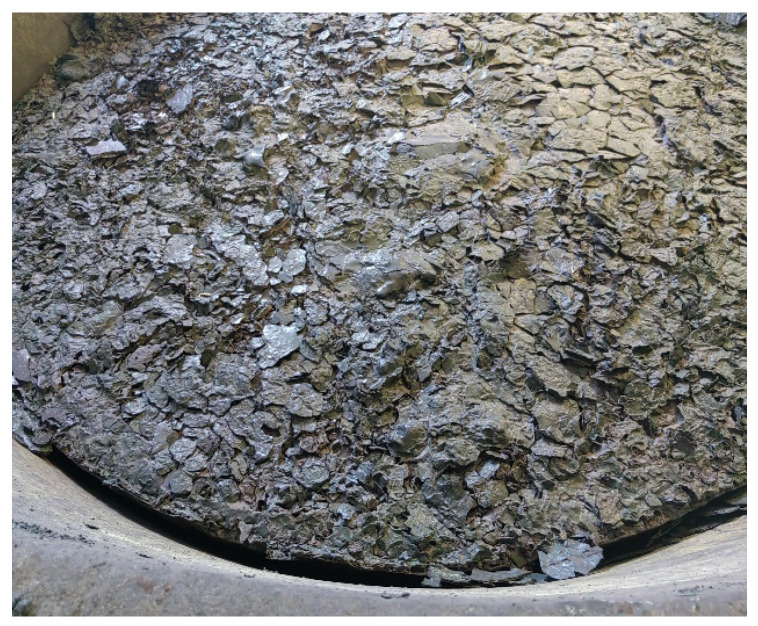
Slag after 168 h of holding in a slag pot under ambient conditions.

After holding the experimental slag in a slag pot for 168 h under ambient outdoor conditions, it remained visually monolithic, without cracks or signs of dust formation (Figure 5). No disintegration was observed during slag dumping at the disposal site.

### 3.5. Microstructure of Slags (SEM Analysis)

**Figure 6:**
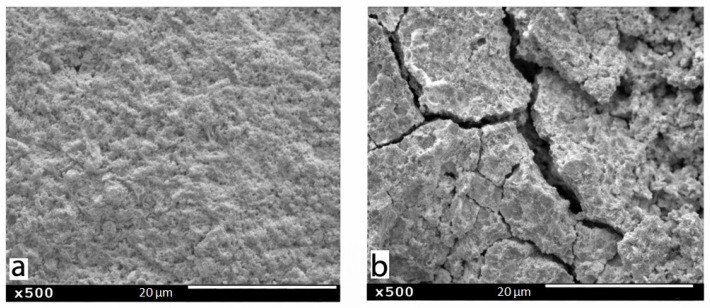
Comparative SEM micrographs of refined ferrochrome slags stabilized with RDBO (**a**) and colemanite (**b**) (×500, scale bar: 20 μm).

To confirm differences in structural stability, the microstructure of slags was examined using scanning electron microscopy (SEM). The results are presented in Figure 6.

It was established that the slag stabilized with remelted borate ore (RDBO) (Figure 6a) is characterized by a dense, homogeneous, and weakly porous structure without visible microcracks. The sample surface exhibits a compact morphology, indicating the formation of stable phases and the absence of significant internal stresses.

In contrast, the slag stabilized with colemanite (Figure 6b) exhibits a pronounced cracked structure. This observation is consistent with previous studies on ferroalloy processing materials, where dense microstructures consisting of silicate and spinel phases were shown to reduce internal stresses and suppress crack formation [20]. Numerous microcracks and regions of structural loosening are observed, indicating the occurrence of silicate disintegration processes associated with the polymorphic transformation of β-Ca_2_SiO_4_ into the γ-form.

The obtained results are in good agreement with the autoclave testing data and confirm that the use of RDBO ensures the formation of a more stable slag microstructure compared to colemanite.

### 3.6. Results of Autoclave Testing

**Figure 7:**
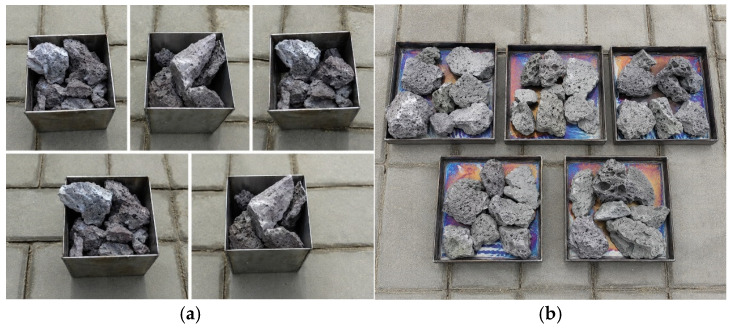
Experimental slag before testing.

Autoclave testing was carried out at a pressure of 400 kPa, a saturated steam temperature of approximately 180 °C, and a holding time of 24 h. Slag pieces with a size of 50–150 mm were tested, using five samples for each stabilization variant (Figure 7 and Table 6). The visual assessment of sample condition was performed according to a four-point scale: 0—intact sample; 1—surface cracks; 2—through cracks; 3—complete disintegration.

**Figure 8:**
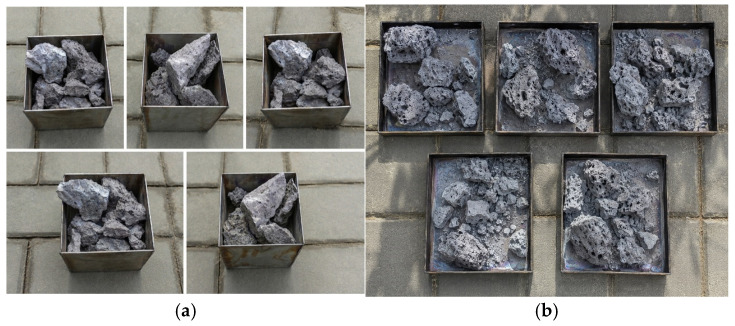
Experimental slag after testing with RDBO (**a**) and colemanite (**b**).

The slag samples stabilized with remelted borate ore (RDBO) (Figure 8a) retained their structural integrity in all five cases (score 0). The average mass loss was 0.19 ± 0.01%, which corresponds to normal moisture evaporation and cannot be considered evidence of structural degradation. In contrast, the slag samples stabilized with colemanite underwent cracking and partial disintegration in all five cases (score 2, Figure 8b). The average mass loss reached 4.76 ± 0.12%, which is more than 25 times higher than that observed for the RDBO-stabilized samples. Such substantial losses are attributed to the mechanical spalling of slag fragments caused by the formation of through-cracks during the β→γ polymorphic transformation of Ca_2_SiO_4_, which is accompanied by a volume expansion of 10–12%.

Thus, the stabilization technology using remelted borate ore (RDBO) ensures long-term structural stability of slag under autoclave exposure, whereas colemanite does not prevent secondary silicate disintegration under prolonged exposure to saturated steam.

### 3.7. Evaluation of the Applicability of Fused Borate Ore (FBO)

The use of fused borate ore (FBO) with a sulfur content of 14.9% was accompanied by intense gas evolution and the release of a pungent odor of sulfur oxides immediately after its addition to the ladle. Due to the resulting unfavorable working conditions and potential risks to equipment, the trials were discontinued. This material was therefore deemed unsuitable for the stabilization of refined ferrochrome slags under the operating conditions of the Aktobe Ferroalloy Plant.

## 4. Discussion

### 4.1. Causes of the Temporary Stabilization Effect of Colemanite

Since 2020, raw colemanite from the Kestelek deposit (Türkiye), containing approximately 38% B_2_O_3_, has been used at the Aktobe Ferroalloy Plant for the stabilization of refined ferrochrome slags. The addition per heat is 115 kg, which corresponds to only about 1% of the total mass of tapped slag (≈10 t). At such a low dosage and with the applied introduction methods (into the furnace or tapping ladle), it is physically impossible to ensure uniform distribution of boron oxide throughout the entire melt volume and to achieve complete reaction of free CaO with the formation of stable borate phases.

In addition, when colemanite is introduced into the furnace, part of the boron transfers into the metal, leading to contamination of ferrochrome and deterioration of its performance characteristics. This necessitates limiting the dosage of the stabilizer, which further reduces the efficiency of stabilization. As a result, the stabilization effect is temporary: after several months of storage, previously stabilized slag undergoes secondary disintegration, as confirmed by both industrial observations and the results of the present study (Figure 5).

Such secondary disintegration leads to the formation of fine particulate matter containing both soluble chromium compounds and boron species, thereby posing a risk of combined contamination of soil and groundwater [15,16].

The obtained results are consistent with data reported in international studies. As reported in [8], colemanite used as the sole stabilizer for ladle furnace slags provides only partial suppression of slag disintegration. At low dosages, the β→γ-Ca_2_SiO_4_ phase transformation may not be completely inhibited, resulting in a limited stabilization effect. The authors attributed this behavior to the non-uniform distribution of boron within the melt and the insufficient contact time between the additive and the slag, which fully corresponds to the conditions of colemanite application at the Aktobe Ferroalloy Plant (115 kg addition, furnace charging).

As reported in [9], complete suppression of the γ-Ca_2_SiO_4_ transformation in synthetic CaO–SiO_2_–MgO–Al_2_O_3_–CrO_x_ slags was achieved when the B_2_O_3_ content reached approximately 2 wt.%. At the same time, the authors noted that exceeding this threshold (>2 wt.%) leads to undesirable chromium redistribution into the Ca_2_SiO_4_ phase and enhances chromium leaching, making excessive boron addition environmentally unacceptable.

In the present study, the B_2_O_3_ content in the stabilized slag ranged from 0.17 to 0.47 wt.%, which is substantially lower than the approximately 2 wt.% B_2_O_3_ reported in [9] for complete suppression of the γ-Ca_2_SiO_4_ transformation in synthetic slags. However, it should be taken into account that industrial refined ferrochrome slags differ substantially from synthetic laboratory slags in terms of the initial Ca_2_SiO_4_ content and overall oxide composition.

### 4.2. Advantages of Using Remelted Borate Ore (RDBO)

The alternative stabilizer proposed in this study—remelted borate ore (RDBO) from the Inder deposit—has several fundamental advantages. First, RDBO undergoes melting, which ensures sulfur removal (the S content is reduced to 0.46% compared to 14.9% in the initial fused ore), thereby eliminating the emission of toxic gases upon contact with the melt. Second, the RDBO addition per heat is 350 kg (approximately three times higher than that of colemanite), allowing the introduction of a sufficient amount of B_2_O_3_ (≈1.7–2.1 kg per ton of slag) for effective binding of free CaO.

Third, RDBO is introduced into the tapping ladle after slag discharge, which completely prevents boron contamination of ferrochrome and avoids negative effects on furnace lining. Although ladle addition requires additional time for mixing, it ensures a locally high boron concentration in the upper slag layers, followed by diffusion and homogenization during subsequent transfer to the slag pot.

#### Comparison of Stabilization Efficiency with Literature Data

A comparative analysis of the results obtained in the present study with published data allows the proposed technology to be positioned within the context of current approaches to metallurgical slag stabilization.

Study [9] reported that the addition of 2 wt.% boron oxide to a synthetic chromium-bearing slag completely suppresses slag disintegration. In the present work, however, slag stability was achieved at a B_2_O_3_ content of only 0.17–0.47 wt.%, which is approximately 4–10 times lower. This difference can be explained by the fundamentally different introduction mechanism: remelted borate ore (RDBO) was added directly into the liquid slag at the tapping temperature (~1510 °C), where boron actively interacted with the melt and formed thermodynamically stable borate phases during crystallization. In the laboratory experiments reported in [9], B_2_O_3_ was introduced into a solid charge mixture, which required significantly higher reagent consumption to achieve a comparable effect. Therefore, high-temperature introduction of RDBO into liquid slag represents not only a technologically, but also a thermodynamically more efficient stabilization approach.

The authors of [8] compared the efficiency of colemanite, perlite, and silicate additives for ladle furnace slags and concluded that the best stabilization performance is achieved through borate modification combined with uniform additive distribution. This conclusion is fully consistent with the recommendations proposed in the present study (Section 4.3), namely the crushing of RDBO to a particle size of 5–10 mm and the implementation of mechanized feeding systems to ensure homogeneous distribution of the additive.

Guo and Li [10] investigated the effect of B_2_O_3_ on the stabilization of high-alumina slags with low MgO content and demonstrated that borate additives form a glassy matrix that suppresses γ-Ca_2_SiO_4_ nucleation. A similar mechanism is supported by the results of the present study: the XRD patterns of slags stabilized with RDBO (Figure 3) show a decrease in the intensity of Ca_2_SiO_4_ peaks accompanied by an increase in CaB_4_O_7_ peaks. Thus, the stabilization mechanism associated with the use of RDBO from the Inder deposit is consistent with the trends established in independent laboratory investigations, confirming its scientific validity and reproducibility.

An important distinction of the proposed approach compared with all the aforementioned studies is its industrial scale and the use of local raw materials (Inder deposit, Western Kazakhstan). All cited investigations [8,9,10] were performed on synthetic or laboratory-prepared slags and did not consider stabilizer behavior under real industrial conditions involving variable slag composition, non-uniform additive distribution, and fluctuating thermal conditions during tapping. This constitutes one of the key scientific novelties of the present work.

**Figure 9:**
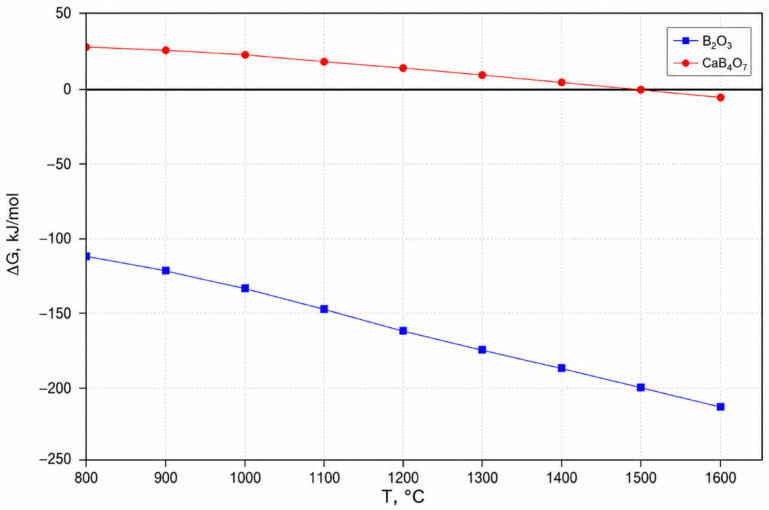
Comparison of the Gibbs free energy for boron reduction from B_2_O_3_ (colemanite) and from CaB_4_O_7_ (RDBO).

At the same time, even the introduction of RDBO into the furnace does not lead to boron contamination of ferrochrome, since boron in RDBO is predominantly present in the form of stable calcium borates (CaB_4_O_7_). As shown in Figure 9, at the smelting temperature (~1600 °C), the reduction of boron from CaB_4_O_7_ by silicon is thermodynamically unfavorable (ΔG is close to zero or positive), whereas boron reduction from free B_2_O_3_, characteristic of raw colemanite, proceeds readily (strongly negative ΔG values). Experimentally, the boron content in ferrochrome produced using RDBO did not exceed 0.001%, whereas with colemanite it reached up to 0.1%. Additional kinetic barriers, together with the binding of B_2_O_3_ by excess CaO present in RDBO, further improve the stability and safety of the process.

The results of the autoclave tests clearly demonstrate the long-term stability of slag stabilized with RDBO (Figure 6), in contrast to slag stabilized with colemanite (Figure 7). The absence of disintegration even after severe exposure to saturated steam at 400 kPa for 24 h indicates that the β-Ca_2_SiO_4_ phase formed during crystallization does not undergo polymorphic transformation into the γ-form.

The stabilization mechanism is governed by competition between the formation of Ca_2_SiO_4_ and CaB_4_O_7_ for available CaO in the slag melt. When processed borate ore (PBO) is introduced into the molten slag at approximately 1510 °C, boron oxide actively reacts with calcium-containing phases, resulting in the formation of thermodynamically stable calcium tetraborate (CaB_4_O_7_). The incorporation of a portion of free CaO into stable borate compounds reduces the thermodynamic activity of CaO in the melt, thereby inhibiting the formation of a critical amount of β-Ca_2_SiO_4_ capable of subsequently transforming into the γ-polymorph.

In addition, borate phases (CaB_4_O_7_) crystallizing along the grain boundaries of Ca_2_SiO_4_ act as a mechanical barrier that restricts volumetric expansion during the β→γ polymorphic transformation and prevents the development of internal stresses [3,5]. This mechanism is supported by the combined experimental and modeling results. Thermodynamic calculations (Table 3) indicate a 20–25% reduction in the Ca_2_SiO_4_ content following the addition of 3.5 kg B_2_O_3_ per tonne of slag. X-ray diffraction analysis (Figure 3) reveals a decrease in the intensity of Ca_2_SiO_4_ peaks accompanied by an increase in CaB_4_O_7_ peaks, while autoclave tests (Table 6) confirm the complete preservation of the structural integrity of the stabilized samples.

Furthermore, the effectiveness of slag stabilization is closely related to the physicochemical properties of the melt, particularly its viscosity and crystallization behavior. It has been shown that increasing slag basicity (CaO/SiO_2_ ratio) and MgO content leads to higher viscosity and crystallization temperatures, which may hinder phase homogenization and mass-transfer processes within the melt [21]. This factor may further limit the effectiveness of colemanite because of its lower dosage and less efficient distribution throughout the slag. In contrast, the higher addition rate of PBO promotes more effective interaction with the slag and enhances the degree of stabilization.

### 4.3. Technical Recommendations for Process Optimization

The conducted trials revealed that the coarse particle size of RDBO (50–150 mm) leads to non-uniform assimilation of boron oxide: in some samples, the B_2_O_3_ content in slag was only 0.01–0.13%, which is below the 0.23–0.35% level recommended by the Ural Institute of Metals [17,22]. To ensure uniform distribution of the stabilizer, the following measures are proposed:

1. Crushing of RDBO to a particle size of 5–10 mm, similar to the colemanite used in current industrial practice. This will increase the contact surface area with the melt and accelerate dissolution.
2. Mechanization of feeding, including the use of pneumatic conveying or a screw feeder to ensure uniform distribution of the material over the slag surface in the ladle.

The implementation of these measures will enable achieving a stable B_2_O_3_ content in slag of at least 0.35%, ensuring complete and long-term stabilization.

### 4.4. Utilization of Stabilized Slag in Road Construction

One of the key advantages of the proposed technology is the possibility of full utilization of stabilized slag in road construction. Previously, in 2003–2004, compositions of road base materials were developed and patented by the authors, in which stabilized refined ferrochrome (RFC) slag acts as a mineral binder [23,24]. According to the patent formulation, the mixture contains (wt.%):

- Slag crushed stone (10–70 mm)—30–40;
- Decomposition products of RFC slag (0–70 mm)—20–30;
- Stabilized RFC slag (0–5 mm or 0–10 mm)—25–35;
- Water—balance.

In such compositions, β-Ca_2_SiO_4_ present in the stabilized slag exhibits hydraulic binding properties, bonding inert aggregates and slag decomposition products into a monolithic, concrete-like structure. This prevents leaching and aeration of fine particles, as confirmed by both laboratory and field tests [24].

Practical implementation of this technology was demonstrated during the construction of a road section between Serov and Sosva (Russia). Slag-based mixtures were laid directly onto waterlogged soil; under the influence of groundwater and atmospheric precipitation, the pavement formed into a monolithic slab that has been operated for more than 10 years without major repairs [25]. This example demonstrates that stabilized refined ferrochrome (RFC) slag is not merely a safe waste material, but a valuable technogenic raw material suitable for the construction of durable road structures.

The slag obtained in the present study, stabilized with RDBO, fully meets the requirements of the aforementioned patents in terms of phase composition (presence of β-Ca_2_SiO_4_) and structural stability. Thus, the proposed technology enables a closed-loop approach: slag stabilization → utilization in road construction → elimination of slag dumps and reduction in the consumption of natural stone materials.

## 5. Conclusions

The conducted study demonstrated that the stabilization of self-disintegrating slags of refined ferrochrome using remelted borate ore (RDBO) from the Inder deposit is an effective and technologically sound solution. Thermodynamic modeling results indicate that the addition of boron oxide leads to the formation of stable borate phases, predominantly CaB_4_O_7_, accompanied by a reduction in the content of dicalcium silicate responsible for silicate disintegration.

X-ray diffraction data experimentally confirm these findings: slags stabilized with RDBO exhibit a decreased fraction of Ca_2_SiO_4_ and the formation of borate compounds, whereas slags stabilized with colemanite retain a significant amount of unstable silicate phases. Scanning electron microscopy results are consistent with phase analysis and show that RDBO promotes the formation of a dense and homogeneous microstructure without signs of cracking, in contrast to colemanite-stabilized slags, which are characterized by pronounced cracking and structural degradation.

It was established that, when using RDBO, a B_2_O_3_ content in slag within the range of 0.17–0.47% is sufficient to prevent disintegration, as confirmed by autoclave testing and long-term observations. In contrast, the use of colemanite in the current technology does not ensure long-term stabilization due to insufficient dosage, non-uniform distribution, and partial transfer of boron into the metal, resulting in secondary slag disintegration during storage.

The proposed stabilization mechanism is based on the binding of free calcium oxide into stable borate compounds and the suppression of the polymorphic transformation of β-Ca_2_SiO_4_ into the γ-form, thereby preventing the development of internal stresses and material degradation. The use of remelted and desulfurized borate ore, combined with increased dosage and ladle addition, ensures not only effective slag stabilization but also eliminates boron contamination of ferrochrome.

The practical significance of this work lies in the possibility of complete utilization of stabilized slags in road construction as a mineral binder, reducing environmental impact and enabling the valorization of technogenic waste. The obtained results can be applied in the development and implementation of industrial technologies for slag stabilization in ferroalloy production.

## Figures and Tables

**Table 1:** Chemical composition of boron-containing materials, wt.%.

Material	CaO	MgO	Al_2_O_3_	FeO	SiO_2_	B_2_O_3_	S
Colemanite	28.0	1.2	0.81	0.42	4.71	39.77	–
RDBO (Sample 1)	56.32	11.22	3.91	0.26	15.75	11.12	1.12
RDBO (Sample 2)	52.99	15.45	2.93	0.66	14.35	14.01	0.46
FBO	36.09	8.83	3.52	0.16	5.75	7.45	14.9

**Table 2:** Chemical composition of refined ferrochrome slag.

Slag Type	Cr_2_O_3_	SiO_2_	CaO	MgO	Al_2_O_3_	FeO	CaO/SiO_2_
RFC slag	5.19	25.92	48.77	12.53	5.87	0.58	1.88

**Table 3:** Results of thermodynamic modeling of phase composition depending on B_2_O_3_ addition and temperature.

B_2_O_3_ Added, kg	0	0.5	1.0	1.5	2.0	2.5	3.0	3.5	4.0	4.5	5.0
25 °C											
Ca_2_SiO_4_, kg	8.599	8.291	7.981	7.672	7.363	7.053	6.744	6.435	6.126	5.816	5.507
Ca_2_SiO_4_, wt.%	11.083	10.686	10.287	9.888	9.490	9.090	8.692	8.294	7.896	7.496	7.098
CaB_4_O_7_, kg	0	0.701	1.403	2.104	2.805	3.507	4.208	4.910	5.610	6.312	7.013
CaB_4_O_7_, wt.%	0	1.817	3.637	5.455	7.272	9.092	10.909	12.729	14.544	16.364	18.181
520 °C											
Ca_2_SiO_4_, kg	8.560	8.273	7.967	7.660	7.352	7.044	6.735	6.427	6.118	5.810	5.501
Ca_2_SiO_4_, wt.%	11.053	10.682	10.287	9.891	9.493	9.095	8.696	8.299	7.900	7.502	7.103
CaB_4_O_7_, kg	0	0.691	1.390	2.090	2.790	3.491	4.191	4.892	5.593	6.293	6.994
CaB_4_O_7_, wt.%	0	1.799	3.618	5.441	7.263	9.088	10.910	12.735	14.559	16.382	18.206
1015 °C											
Ca_2_SiO_4_, kg	8.508	8.287	8.0417	7.790	7.538	7.287	7.038	6.790	6.545	6.303	6.064
Ca_2_SiO_4_, wt.%	10.610	10.334	10.028	9.714	9.400	9.087	8.776	8.467	8.162	7.860	7.562
CaB_4_O_7_, kg	0	0.467	1.020	1.584	2.151	2.717	3.280	3.839	4.393	4.941	5.483
CaB_4_O_7_, wt.%	0	1.563	3.414	5.302	7.200	9.095	10.979	12.850	14.705	16.539	18.353
1510 °C											
Ca_2_SiO_4_, kg	8.478	8.307	8.111	7.909	7.706	7.504	7.303	7.105	6.910	6.717	6.528
Ca_2_SiO_4_, wt.%	10.267	10.060	9.822	9.578	9.332	9.087	8.844	8.604	8.368	8.134	7.905
CaB_4_O_7_, kg	0	0.313	0.738	1.181	1.630	2.078	2.524	2.965	3.400	3.830	4.253
CaB_4_O_7_, wt.%	0	1.366	3.221	5.155	7.114	9.069	11.016	12.941	14.839	16.716	18.562
2005 °C											
Ca_2_SiO_4_, kg	8.419	8.201	7.978	7.753	7.530	7.308	7.089	6.873	6.661	6.452	6.246
Ca_2_SiO_4_, wt.%	10.457	10.186	9.909	9.630	9.353	9.077	8.805	8.537	8.274	8.014	7.758
CaB_4_O_7_, kg	0	0.278	0.697	1.149	1.613	2.081	2.549	3.015	3.477	3.933	4.384
CaB_4_O_7_, wt.%	0	1.200	3.007	4.958	6.960	8.979	10.998	13.009	15.003	16.970	18.916
2500 °C											
Ca_2_SiO_4_, kg	8.357	8.071	7.799	7.533	7.272	7.015	6.763	6.514	6.269	6.028	5.791
Ca_2_SiO_4_, wt.%	10.795	10.426	10.075	9.731	9.394	9.062	8.736	8.415	8.098	7.787	7.481
CaB_4_O_7_, kg	0	0.255	0.682	1.160	1.661	2.173	2.690	3.209	3.728	4.244	4.757
CaB_4_O_7_, wt.%	0	1.038	2.777	4.723	6.763	8.848	10.953	13.066	15.180	17.281	19.370

**Table 4:** Chemical composition of slags obtained using RDBO, wt.%.

Sample No.	Sampling Location	Cr_2_O_3_	SiO_2_	CaO	MgO	Al_2_O_3_	FeO	B_2_O_3_
251048	Furnace	9.19	22.92	47.99	12.46	5.14	2.29	0.01
200172	Ladle	9.18	22.50	46.60	12.68	5.59	2.17	0.21
251049	Furnace	9.37	24.58	46.75	11.25	5.39	2.64	0.01
200173	Ladle	9.54	22.03	41.28	17.19	6.20	3.73	0.13
251050	Furnace	11.49	22.49	44.74	12.88	5.57	2.81	0.10
200174	Ladle	9.88	24.50	46.21	11.28	5.30	2.82	0.01

**Table 5:** Chemical composition of stabilized slag after holding, wt.%.

Sample No.	Cr_2_O_3_	SiO_2_	CaO	MgO	Al_2_O_3_	FeO	B_2_O_3_
200209	11.18	22.18	47.85	11.91	5.36	1.50	0.17
200210	6.78	23.33	50.36	11.81	5.39	2.31	0.18
501111	9.15	24.44	44.66	13.39	5.77	2.57	0.47

**Table 6:** Results of autoclave testing of stabilized slags.

No.	Stabilizer Type	Mass Before, g	Mass After, g	Mass Loss, %	Score (0–3)	Sample Condition
Samples with RDBO (Remelted Borate Ore, Inder deposit)						
1	RDBO	245.3	244.8	0.20	0	Intact, no cracks
2	RDBO	312.7	312.1	0.19	0	Intact, no cracks
3	RDBO	278.5	278.0	0.18	0	Intact, no cracks
4	RDBO	301.2	300.6	0.20	0	Intact, no cracks
5	RDBO	267.4	266.9	0.19	0	Intact, no cracks
Average ± SD				0.19 ± 0.01	0	100% of samples—score 0
Samples with colemanite (Kestelek deposit, Türkiye)						
1	Colemanite	280.1	267.3	4.57	2	Through cracks, partial disintegration
2	Colemanite	295.4	281.0	4.87	2	Through cracks, partial disintegration
3	Colemanite	310.2	295.1	4.87	2	Through cracks, partial disintegration
4	Colemanite	272.8	259.9	4.73	2	Through cracks, partial disintegration
5	Colemanite	288.6	274.8	4.78	2	Through cracks, partial disintegration
Average ± SD				4.76 ± 0.12	2	100% of samples—score 2
Summary comparison						
Parameter		RDBO		Colemanite		Ratio
Average mass loss, %		0.19		4.76		×25
SD of mass loss, %		±0.01		±0.12		–
Average score (0–3)		0		2		–
Intact samples, %		100%		0%		–
Disintegrated samples, %		0%		100%		–

## Data Availability

The data presented in this study are available within the article.

## Abbreviations

The following abbreviations are used in this manuscript:

RFC	Refined Ferrochrome
RDBO	Remelted and Desulfurized Borate Ore
FBO	Fused Borate Ore
